# Supplementary material for: Electrostatic Adsorption of Dense AuNPs onto Silica Core as High-Performance SERS Tag for Sensitive Immunochromatographic Detection of Streptococcus pneumoniae
Source: Pathogens. 2023 Feb 15;12(2):327. doi: 10.3390/pathogens12020327 (PMC9965993; doi:10.3390/pathogens12020327)
Supplement: Supplementary file 1 [file pathogens-12-00327-s001.zip › pathogens-2192852-supplementary.pdf]

pathogens

S1. Experimental section

S1.1 Instruments and detection parameters for SERS detection

The structure and morphology of SERS tags were investigated by transmission electron microscope (TEM, Philips Tecnai G2 F20) and scanning electron microscope (SEM, JEOL JSM-7001F). The TEM images of bacteria were obtained using a Hitachi H-7650 microscope operating at 80 kV. The zeta potential values and of UV-vis absorption spectra the fabricated SiO_2@20Au NPs were measured with a nano ZS90 zeta instrument (Malvern, UK) and a UV-2600 spectrometer (Shimadzu, Japan), respectively. The SERS detection was standardized by setting uniform detection conditions and data recording was performed using a Renishaw InVia Qontor Raman spectrometer with 785 nm laser excitation. The laser power, integration time and light spot of the Raman instrument were set at 5 mW, 5 s and ~100 μm, respectively. To reduce experimental error, twenty random tests were randomly measured on the test zone for each sample and the obtained SERS spectra were averaged for subsequent analysis. For validation of the result, different groups of samples were measured independently and all the experiments were performed three times. The SERS mapping images of the T lines of ICA were obtained with laser power and acquisition time set at 1% and 1 s, respectively. For each test zone, a 40 μm (x axis) and 110 μm (y axis) range (total 176 pixels) was scanned with a pixel size of 5 μm × 5 μm by using a computer-controlled x–y translational stage.

S1.2 Preparation of AuNP-based ICA

First, we prepared 40 nm Au NPs using a conventional trisodium citrate reduction method. Second, the pH of 10 μg of *S. pneumoniae* antibodies were adjusted to 9 with 0.2 M K_2CO_3 and incubated with 1 mL 40 nm AuNP (pH 8-9) for 15min. Then, 100 μL of BSA solution (10 mg/mL) was added to block the unreacted sites of AuNPs. The as-prepared immuno-AuNPs were separated by centrifugation (4000 rpm, 6 min), and resuspended with 200 μL of storage solution (10 mM PB solution containing 1% BSA (w/v), 0.1% PVP (w/v), 10% sucrose (w/v), and 0.05% Tween-20 (v/v)). Finally, the antibody-conjugated AuNPs were directly mixed with the bacteria sample and dropped onto the sample pad of ICA.

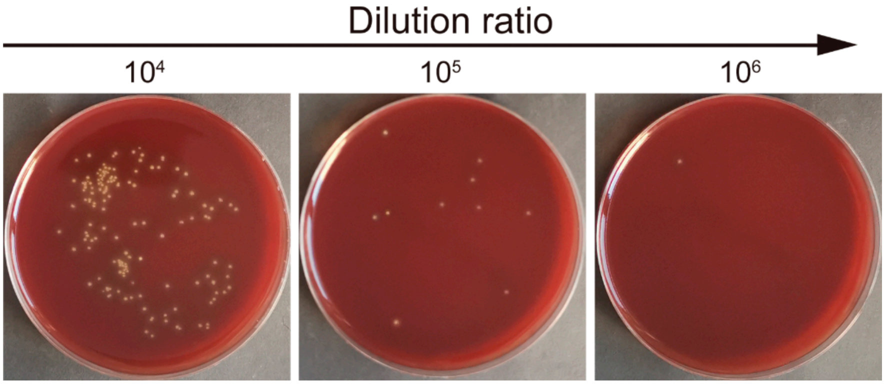

Figure S1. Plate counting results for *S. pneumoniae.*

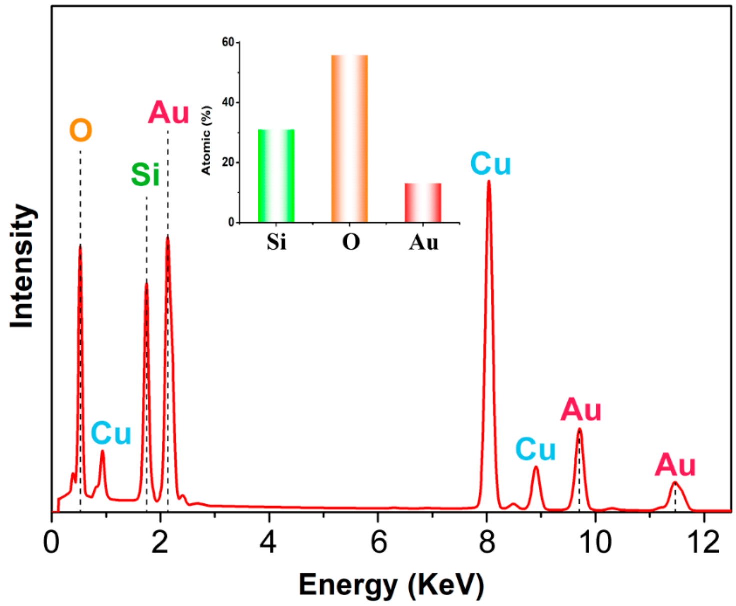

Figure S2. EDS data from a single SiO_2@20Au tag. The Cu signal is from the Cu grids of the TEM sample. The inset shows the element compositions in SiO_2@20Au structure.

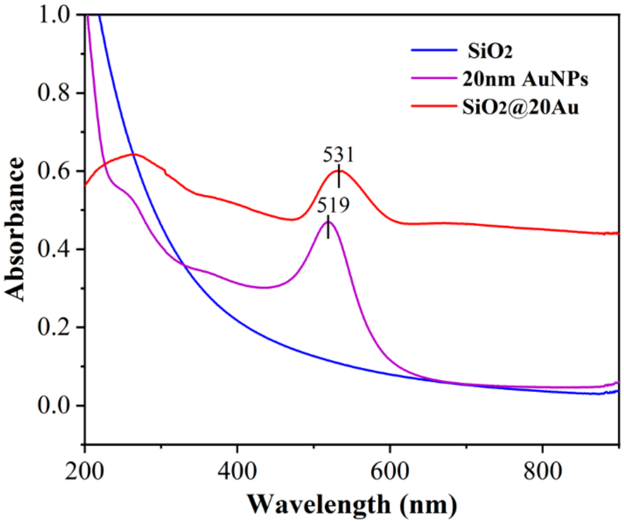

Figure S3. UV-visible spectra of SiO_2, 20 nm AuNPs, and SiO_2@20Au.

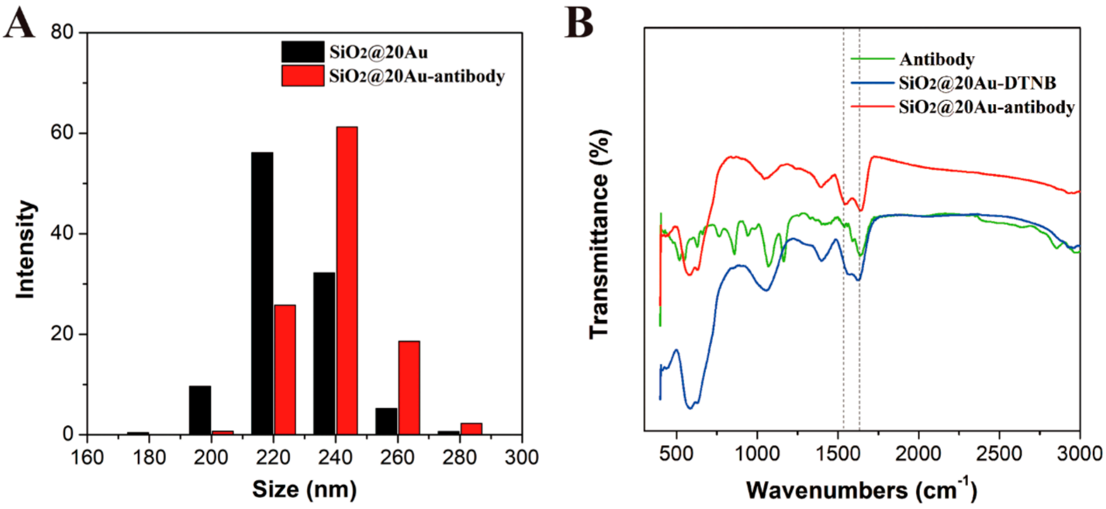

Figure S4. (A) DLS distributions of SiO_2@20Au (black) and antibody-modified SiO_2@20Au tags (red). The DLS data reveals that the average diameter of SiO_2@20Au increased from 220 nm to 240 nm after antibody conjugation. **(B)** FTIR spectra of SiO_2@20Au (blue line), anti-*S. pneumoniae* antibody (green line), and antibody-modified SiO_2@20Au tags (red line). The characteristic absorption peaks corresponding to protein amide bands I (1641 cm^{-1}) and II (1530 cm^{-1}) appearing in immuno-SiO_2@20Au reveals the success of coupling.

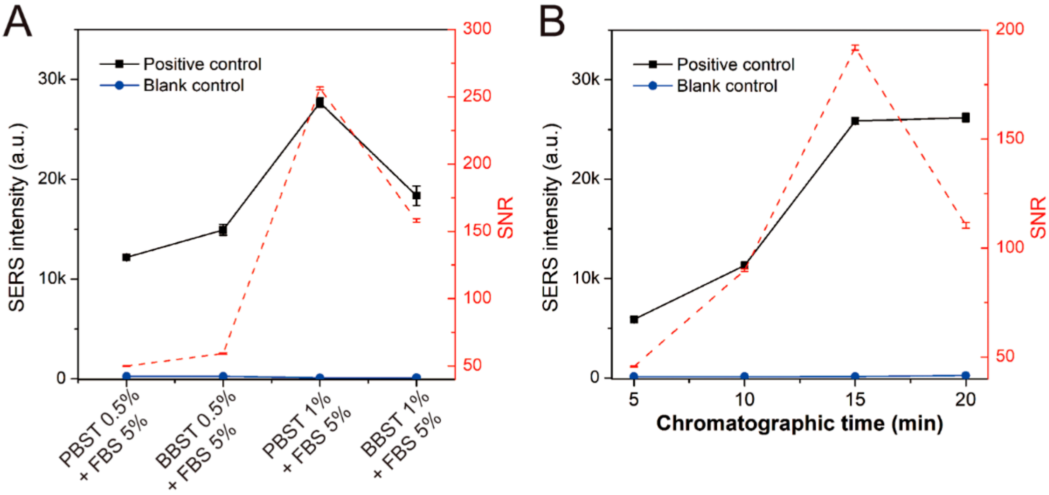

Figure S5. Optimization of **(A)** running buffer and **(B)** chromatographic time for SiO_2@20Au-based SERS-ICA. The error bars indicate standard deviations calculated from three measurements.

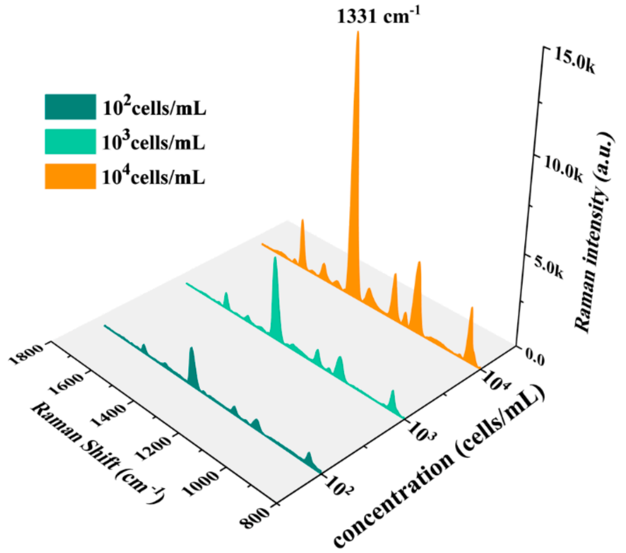

Figure S6. The corresponding SERS signals on the test line of SiO_2@20Au-ICA strips for human sputum samples with different concentrations (10^4, 10^3, 10^2 cells/mL) of *S. pneumoniae.*

Table S1. Recovery efficiencies of *S. pneumoniae* spiked in real human sputum samples.

Sample	Strain	Spiked (cells/mL)	Detected (cells/mL)	Recovery (%)	CV (%)
Sputum	*S. pneumoniae*	1×10^4	8926	89.2	6.7
		1×10^3	1246	124.6	8.9
		1×10^2	109	109.0	11.3
